# Cytoprotective effects of mild plasma-activated medium against oxidative stress in human skin fibroblasts

**DOI:** 10.1038/srep42208

**Published:** 2017-02-07

**Authors:** Minori Horiba, Tetsuro Kamiya, Hirokazu Hara, Tetsuo Adachi

**Affiliations:** 1 Laboratory of Clinical Pharmaceutics, Gifu Pharmaceutical University, Gifu 501-1196, Japan

## Abstract

Non-thermal atmospheric pressure plasma (NTAPP) has recently been applied to living cells and tissues and has emerged as a novel technology for medical applications. NTAPP affects cells not only directly, but also indirectly with previously prepared plasma-activated medium (PAM). The objective of this study was to demonstrate the preconditioning effects of “mild PAM” which was prepared under relatively mild conditions, on fibroblasts against cellular injury generated by a high dose of hydrogen peroxide (H_2_O_2_). We observed the preconditioning effects of mild PAM containing approximately 50 μM H_2_O_2_. Hydrogen peroxide needs to be the main active species in mild PAM for it to exert preconditioning effects because the addition of catalase to mild PAM eliminated these effects. The nuclear translocation and recruitment of nuclear factor erythroid 2-related factor 2 (Nrf2) to antioxidant response elements (ARE) in *heme oxygenase 1 (HO-1)* promoters and the up-regulation of HO-1 were detected in fibroblasts treated with mild PAM. The addition of ZnPP, a HO-1-specific inhibitor, or the knockdown of Nrf2 completely abrogated the preconditioning effects. Our results demonstrate that mild PAM protects fibroblasts from oxidative stress by up-regulating HO-1, and the H_2_O_2_-induced activation of the Nrf2-ARE pathway needs to be involved in this reaction.

Plasma is the fourth state of matter and is defined as an ionized gas. According to its gas temperature, plasma has been divided into thermal and non-thermal plasma. Non-thermal atmospheric pressure plasma (NTAPP) has recently been applied to living cells and tissues^1^ and has emerged as a novel technology for clinical medicine, for example, wound healing^2^, blood coagulation^3^, sterilization^4^, and cancer treatments^1^,^5^,^6^. In these studies on its clinical application, a low dose of NTAPP was reported to induce the proliferation of cells, whereas a high dose induced apoptosis^7^. NTAPP contains variable compositions of ultraviolet light, free electrons, and charged particles as well as reactive oxygen species (ROS) such as the hydroxyl radical (•OH), superoxide (O_2_^−^), ozone (O_3_), and hydrogen peroxide (H_2_O_2_), and reactive nitrogen species (RNS) including nitric oxide (NO•) and peroxynitrite (ONOO^−^)^8^,^9^. Most of the functions of NTAPP are involved in the activities of ROS and/or RNS^10^,^11^,^12^.

ROS affect several signaling pathways in cells such as protein kinases, transcription factors, and cellular signaling proteins^13^. High levels of intracellular ROS are known to be harmful because they trigger significant oxidative damage in cellular macromolecules, such as nucleotides, lipid, and proteins, whereas low levels of intracellular ROS are necessary for a number of important physiological functions including intracellular signal transduction and host defenses against microorganisms^14^. “Preconditioning” is a phenomenon in which brief oxidative stress induced by low levels of ROS may result in protection against a subsequent stronger stress. The Kelch-like ECH-associated protein 1 (Keap1)-nuclear factor erythroid 2-related factor 2 (Nrf2)-antioxidant response element (ARE) signaling pathway is one of the important ROS-induced physiological functions maintaining cellular redox homeostasis^15^. Under normal conditions, Nrf2 is rapidly trapped by Keap1 in the cytoplasm and degraded by ubiquitin-dependent proteolysis. On the other hand, with increases in the levels of electrophilic compounds including ROS, Keap1 is inactivated via the oxidation of its cysteine residues and dissociates Nrf2. Therefore, Nrf2 is stabilized and translocated to the nucleus, and then binds to ARE, a *cis*-regulatory DNA sequence located in the promoter of target genes encoding phase II detoxifying enzymes, and cytoprotective proteins including heme oxygenase 1 (HO-1)^16^,^17^. Among various ROS, H_2_O_2_ is known to activate Nrf2^18^,^19^,^20^.

NTAPP has been reported to affect cells not only directly, but also indirectly with previously prepared plasma-activated medium (PAM)^6^,^21^,^22^,^23^. The relatively short-lived ROS and RNS produced in medium by NTAPP irradiation are converted to other relatively long-lived species such as H_2_O_2_, nitrite/nitrate (NOx), and other unknown species^21^,^24^,^25^. PAM has recently been shown to induce apoptosis in cancer cells^6^,^25^,^26^,^27^,^28^,^29^,^30^. On the other hand, the effects of PAM prepared under relatively mild conditions, termed mild PAM, on normal cells are currently unclear. The stimulation of Phase II antioxidant pathways in keratinocytes by PAM has recently been reported^31^.

The objective of this study was to demonstrate the preconditioning effects of mild PAM on fibroblasts against cellular injury generated by a high dose of H_2_O_2_ as an oxidative stress inducer and elucidate the involvement of the Keap1-Nrf2-ARE signaling pathway in this cytoprotection.

## Results

### Reductions in H_2_O_2_-induced cell injury by the pretreatment with mild PAM

**Figure 1:**
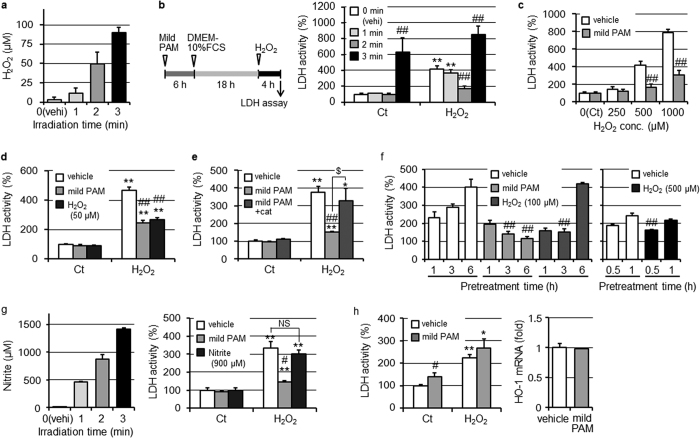
Pretreatment with mild PAM reduced H_2_O_2_-induced cell injury. (**a**) Concentrations of H_2_O_2_ in mild PAM prepared by the irradiation of NTAPP for the indicated times. (**b**, left) Experimental procedure; fibroblasts were pretreated with mild PAM for 6 h in a CO_2_ incubator. After the exchange of mild PAM to DMEM-10% FCS, fibroblasts were cultured for 18 h in a CO_2_ incubator. Fibroblasts were then treated with 500 μM H_2_O_2_ in DMEM for 4 h in a CO_2_ incubator, followed by an assay for LDH released into conditioned medium. (**b**, right) Fibroblasts were pretreated with mild PAM prepared by the irradiation of NTAPP for the indicated times. Control (Ct) fibroblasts were incubated with DMEM for 4 h instead of 500 μM H_2_O_2_ in DMEM. (**c**) After the pretreatment with mild PAM or DMEM (vehicle), and followed by the incubation with DMEM-10% FCS, fibroblasts were treated with DMEM with or without 250, 500, and 1000 μM H_2_O_2_ for 4 h, followed by the LDH assay. (**d**) Fibroblasts were pretreated with mild PAM, 50 μM H_2_O_2_ in DMEM, or DMEM (vehicle) for 6 h, followed by the procedure described above. (**e**) Fibroblasts were treated with mild PAM in the presence or absence of catalase (50 U/mL) or DMEM (vehicle) for 6 h, followed by the procedure described above. (**f**) Fibroblasts were treated with mild PAM, 100 μM H_2_O_2_ in DMEM, 500 μM H_2_O_2_ in DMEM, or DMEM (vehicle) for the indicated times, followed by the procedure described above. (**g**, left) Concentrations of nitrite in mild PAM prepared by irradiation with NTAPP for the indicated times. (**g**, right) Fibroblasts were pretreated with mild PAM, 900 μM nitrite in DMEM, or DMEM (vehicle) for 6 h. (**h**, left) A549 cells were pretreated with mild PAM or DMEM (vehicle) for 6 h, followed by the procedure described above. (**h**, right) A549 cells were treated with mild PAM or DMEM (vehicle) for 6 h, and HO-1 mRNA levels were then measured. Data are shown as the mean ± SD (n = 3), *p < 0.05, **p < 0.01 vs. the control, ^#^*p* < 0.05, ^##^*p* < 0.01 vs. vehicle, ^$^*p* < 0.05, NS not significant.

NTAPP irradiation produced H_2_O_2_ in DMEM in irradiation time-dependent manners under our experimental conditions, as shown in Fig. 1a. In the present study, fibroblasts were treated with mild PAM for 6 h and then incubated with culture medium for 18 h. Fibroblasts were then treated with 500 μM H_2_O_2_ as strong oxidative stress for 4 h, as shown in Fig. 1b left panel, except in specifically described experiments. Cell injury induced by the treatment with 500 μM H_2_O_2_ was significantly suppressed by the pretreatment with mild PAM prepared by plasma irradiation for 2 min, whereas mild PAM prepared by irradiating for 1 min did not affect cell injury, as shown in Fig. 1b right panel. On the other hand, (mild) PAM prepared by irradiating for 3 min induced cell injury, even without the H_2_O_2_ treatment. Fibroblasts pretreated with mild PAM significantly resisted cytotoxicity induced by H_2_O_2_ at concentrations of 500 or 1000 μM (Fig. 1c). H_2_O_2_ is known to be the main active species in PAM^24^,^25^,^26^,^27^, and mild PAM prepared by an irradiation time for 2 min contained approximately 50 μM H_2_O_2_, as shown in Fig. 1a. The pretreatment with 50 μM H_2_O_2_-supplemented DMEM provided a similar degree of suppression against H_2_O_2_-induced cellular injury to mild PAM (Fig. 1d). Moreover, fibroblasts pretreated with mild PAM supplemented with catalase (50 U/ml) did not resist cell injury (Fig. 1e). The preconditioning effects of mild PAM depend on its H_2_O_2_ concentration and pretreatment time. Cell injury was significantly and maximally suppressed by the pretreatment with mild PAM for 6 h and followed by 3 h, as shown in Fig. 1f left panel. On the other hand, 100 μM H_2_O_2_-supplemented DMEM, the H_2_O_2_ concentration of which was the same as that of (mild) PAM prepared by NTAPP irradiation for 3 min, exerted preconditioning effects by the pretreatment for 3 h. However, cell injury was induced by the pretreatment for 6 h. The pretreatment with 500 μM H_2_O_2_-supplemented DMEM, the H_2_O_2_ concentration of which was similar to that of PAM used in previous studies^25^,^27^,^28^, for 30 min suppressed the release of LDH (Fig. 1f right). However, cells detached from the culture dish following the pretreatment with 500 μM H_2_O_2_-supplemented DMEM for 3 h.

Another main species in mild PAM is NOx, and NTAPP irradiation for 2 min produced approximately 900 μM nitrite as NOx (Fig. 1g left). However, the pretreatment with 900 μM nitrite-supplemented DMEM did not reduce cell injury (Fig. 1g right).

We previously reported that a PAM treatment effectively induced apoptosis in A549 human lung adenocarcinoma epithelial cells^25^,^27^,^28^. When A549 cells were treated with mild PAM according to the protocol shown in Fig. 1b left, mild PAM led to slightly greater cell injury than that with the vehicle, as shown in Fig. 1h left panel.

### Induction of HO-1 by the treatment with mild PAM

**Figure 2:**
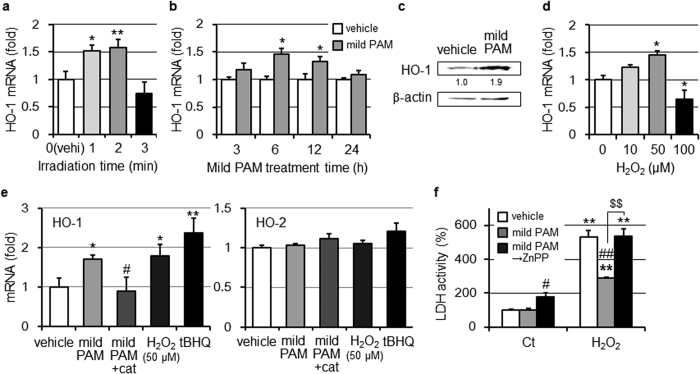
Mild PAM induced HO-1 expression. (**a**) Fibroblasts were treated with mild PAM prepared by the irradiation of NTAPP for the indicated times, and HO-1 mRNA levels were measured. Data are shown as the mean ± SD (n = 3), *p < 0.05, **p < 0.01 vs. vehicle. (**b**) Fibroblasts were treated with mild PAM or DMEM (vehicle) for the indicated times, and HO-1 mRNA levels were measured. Data are shown as the mean ± SD (n = 3), *p < 0.05 vs. vehicle. (**c**) Fibroblasts were treated with mild PAM or DMEM (vehicle) for 6 h and then cultured in DMEM-10%FCS for 18 h in a CO_2_ incubator. HO-1 protein levels were then measured by Western blotting. Values are fold changes from vehicle. (**d**) Fibroblasts were treated with the indicated concentration of H_2_O_2_ in DMEM for 6 h, and HO-1 mRNA levels were measured. Data are shown as the mean ± SD (n = 3), *p < 0.05 vs. vehicle. (**e**) Fibroblasts were treated with mild PAM in the presence or absence of catalase (50 U/ml), 50 μM H_2_O_2_, 20 μM tBHQ, or DMEM (vehicle) for 6 h, and the mRNA levels of HO-1 (left) or HO-2 (right) were then measured. Data are shown as the mean ± SD (n = 3), *p < 0.05, **p < 0.01 vs. vehicle, ^#^*p* < 0.05 vs. mild PAM only. (**f**) After the pretreatment with mild PAM or DMEM (vehicle) for 6 h, fibroblasts were treated with DMEM-10% FCS for 18 h and then 500 μM H_2_O_2_ for 4 h in the presence or absence of 10 μM ZnPP, followed by the LDH cytotoxic test. Control (Ct) fibroblasts were incubated with DMEM for 4 h instead of 500 μM H_2_O_2_ in DMEM. Data are shown as the mean ± SD (n = 3), **p < 0.01 vs. the control, ^#^*p* < 0.05, ^##^*p* < 0.01 vs. vehicle, ^$$^*p* < 0.01.

In order to elucidate the effects of mild PAM on the expression of HO-1, fibroblasts were treated with mild PAM for 6 h. Mild PAM prepared by NTAPP irradiation for 1 or 2 min increased the expression of HO-1 mRNA (Fig. 2a). On the other hand, (mild) PAM prepared by irradiation for 3 min decreased HO-1 mRNA levels. The mild PAM treatment for 6 h maximally enhanced the expression of HO-1 mRNA (Fig. 2b). The protein level of HO-1 in mild PAM-treated cells also increased (Fig. 2c). HO-1 mRNA levels were significantly increased by the treatment with 50 μM H_2_O_2_-supplemented DMEM, but were only moderately increased by that with 10 μM H_2_O_2_-supplemented DMEM. HO-1 mRNA levels were decreased by the treatment with 100 μM H_2_O_2_-supplemented DMEM (Fig. 2d). Mild PAM supplemented with catalase did not induce the expression of HO-1 mRNA, while 50 μM H_2_O_2_-supplemented DMEM achieved the similar up-regulation of HO-1 mRNA to mild PAM. *tert*-butylhydroquinone (tBHQ), a known HO-1 enhancer, up-regulated HO-1 mRNA (Fig. 2e, left). On the other hand, the mRNA of HO-2, a constitutive enzyme, was not induced by the treatment with mild PAM (Fig. 2e right). These results suggest that the ability of mild PAM to induce HO-1 depends on H_2_O_2_. In an attempt to clarify whether HO-1 induced by the mild PAM treatment confers cytoprotection against toxicity induced by a high dose of H_2_O_2_, fibroblasts were cultured in the presence of the HO-1 inhibitor ZnPP (10 μM) after a pretreatment with mild PAM. ZnPP eliminated the protective effects of mild PAM against H_2_O_2_-induced cell injury (Fig. 2f).

The treatment of A549 cells with mild PAM did not affect HO-1 mRNA levels (Fig. 1h right panel).

### Enhancement of the ARE-binding activity of Nrf2 in mild PAM-treated fibroblasts

**Figure 3:**
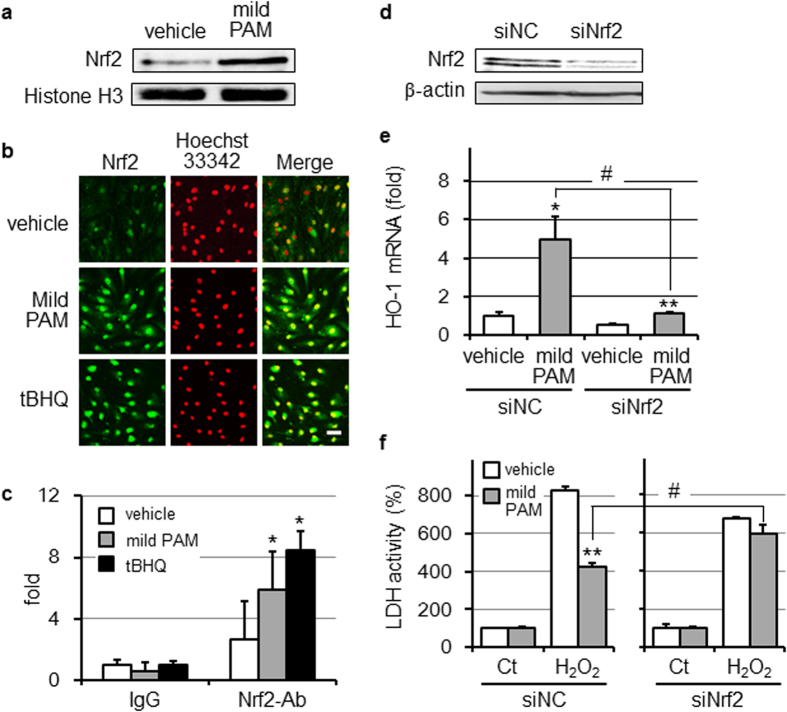
Mild PAM enhanced Nrf2 signaling. (**a**) Fibroblasts were treated with mild PAM or DMEM (vehicle) for 6 h and their nuclear fractions were then isolated and subjected to Western blotting for Nrf2. (**b**) The nuclear translocation of Nrf2 was assessed by immunofluorescence staining. Fibroblasts were treated with mild PAM, tBHQ (20 μM), or DMEM (vehicle) for 6 h. Cells were fixed and labeled with an anti-Nrf2 antibody, as described in the Materials and methods. Cell nuclei were labeled with Hoechst 33342. Scale bar, 50 μm. (**c**) The ARE binding activity of Nrf2 was measured using the ChIP assay. Fibroblasts were treated with mild PAM, tBHQ (20 μM), or DMEM (vehicle) for 6 h. Their chromatins were cross-linked and immunoprecipitated with IgG or an anti-Nrf2 antibody (Ab), and DNAs were amplified using an ARE-specific primer. Data are shown as the mean ± SD (n = 3), *p < 0.05 vs. vehicle. (**d**) After siNrf2 and the siNC were transfected into fibroblasts for 24 h in a CO_2_ incubator, whole cell extracts were prepared and followed by Western blotting for Nrf2. (**e**) After transfection of siNrf2 or siNC, cells were treated with mild PAM or DMEM (vehicle) for 6 h and HO-1 mRNA levels were then measured. Data are shown as the mean ± SD (n = 3), *p < 0.05, **p < 0.01 vs. vehicle, ^#^*p* < 0.05. (**f**) After transfection of siNrf2 or siNC, cells were treated with mild PAM or DMEM (vehicle) for 6 h, followed by cultivation for 18 h, a treatment with or without 500 μM H_2_O_2_ for 4 h, and the LDH assay. Control (Ct) fibroblasts were incubated with DMEM for 4 h instead of 500 μM H_2_O_2_ in DMEM. Data are shown as the mean ± SD (n = 3), **p < 0.01 vs. vehicle, ^#^*p* < 0.05.

Nrf2 is a major transcription factor that binds to ARE in the promoter region of some antioxidative enzymes. When fibroblasts were treated with mild PAM for 6 h, the nuclear translocation of Nrf2 was detected (Fig. 3a). This Nrf2 nuclear translocation was also observed by fluorescence imaging with a confocal microscopic analysis (Fig. 3b). In addition, ChIP assays showed an increase in Nrf2 binding to ARE in the *HO-1* promoter (Fig. 3c). In order to elucidate whether Nrf2-mediated transcriptional activation is involved in the induction of HO-1 in response to mild PAM, we transfected Nrf2-specific siRNA (siNrf2) or control siRNA into fibroblasts and treated these cells with mild PAM for 6 h. We confirmed that the transfection of siNrf2 caused a marked reduction in Nrf2 in fibroblasts (Fig. 3d). siNrf2 attenuated the induction of HO-1 mRNA by the treatment with mild PAM (Fig. 3e). Moreover, mild PAM-induced cytoprotection against toxicity induced by a high dose of H_2_O_2_ was significantly reduced by the transfection of siNrf2 (Fig. 3f).

## Discussion

ROS and/or RNS or their derived species are generally considered to be the main bioactive components of PAM^8^,^9^,^10^,^11^,^12^. We previously reported that a PAM treatment effectively induced apoptosis in A549 lung adenocarcinoma cells^25^,^27^,^28^. H_2_O_2_ and/or its derived •OH disturb the mitochondrial-nuclear network in cells. PAM used in previous studies contained approximately 500 to 600 μM H_2_O_2_, a concentration that was about 10-fold higher than that in mild PAM used in this study. On the other hand, preconditioning effects of H_2_O_2_ have been reported in experiments with cultured cells^32^,^33^,^34^ and perfusion experiments using isolated rat hearts^35^. Low concentrations of H_2_O_2_, between approximately 10 to 100 μM, exerted effects in these studies^31^,^32^,^33^,^35^, whereas a high dose of H_2_O_2_ (500 and 1000 μM) was used to induce strong oxidative stress^34^. As shown in Fig. 1, we observed preconditioning effects by mild PAM prepared using NTAPP irradiation for 2 min, which contained approximately 50 μM H_2_O_2_, but not by PAM prepared using NTAPP irradiation for 1 min (containing approximately 10 μM H_2_O_2_). PAM prepared using NTAPP irradiation for 3 min (containing approximately 100 μM H_2_O_2_) showed cell toxicity. H_2_O_2_ needs to be the main active species in mild PAM for it to exert preconditioning effects because the addition of catalase to mild PAM eliminated these effects (Fig. 1e). The preconditioning effects of mild PAM depend on its H_2_O_2_ concentration and pretreatment time. We examined whether the pretreatment with H_2_O_2_ at a higher concentration (100 or 500 μM) for a shorter treatment time (0.5 to 3 h) exerted preconditioning effects. However, the pretreatment with mild PAM (about 50 μM H_2_O_2_) showed the safest and most reliable preconditioning effects (Fig. 1f). Mild PAM containing tens of μM of H_2_O_2_ may exert preconditioning effects; however, sensitivity to H_2_O_2_ differs among cells.

Nitrite, another main species in mild PAM, did not exert preconditioning effects (Fig. 1g). Nitrite by itself has been suggested to not play a role in the antitumor effects of PAM^25^,^36^. However, nitrite may affect the potency of PAM and mild PAM because the cancer cell-killing ability of H_2_O_2_ is reported to be synergistically enhanced by nitrite^36^.

Nrf2-mediated HO-1 induction was previously shown to play crucial roles in the anti-oxidative defense system^34^,^35^,^36^,^37^,^38^,^39^. HO catalyzes the degradation of heme to carbon monoxide, a vasoactive gas; biliverdin, an antioxidant; and free iron^40^. The HO system possesses antioxidative and anti-apoptotic properties, and may influence cell proliferation, differentiation, and migration^41^. Therefore, we postulated that the Nrf2-HO system may be involved in mild PAM-induced preconditioning effects. Among the three HO isoforms (HO-1, HO-2, and HO-3), HO-1 is the only inducible enzyme that plays a critical role in cytoprotection against oxidative stress, inflammation, and other noxious stimuli^37^. In the present study, mild PAM elevated HO-1 mRNA and protein levels, but not those of HO-2 mRNA (Fig. 2e). Moreover, 50 μM H_2_O_2_ significantly increased HO-1 mRNA levels (Fig. 2d). The induction of HO-1 mRNA was not observed when fibroblasts were treated with catalase-supplemented mild PAM (Fig. 2e, left). These results suggest that H_2_O_2_ in mild PAM is critical for the mild PAM-induced expression of HO-1. However, we cannot rule out the possibility that a mechanism other than the induction of HO-1 is involved in the preconditioning effects of the mild PAM pretreatment, and other unknown species in mild PAM may play a role in the induction of HO-1 because mild PAM prepared using NTAPP irradiation for 1 min increased HO-1 mRNA levels, but did not affect cell injury (Fig. 1b), and the induction of HO-1 by 10 μM H_2_O_2_ was moderate (Fig. 2d). The roles of HO-2 and HO-3 in cells currently remain unclear. HO-2 mRNA levels are low in human dermal fibroblasts, but high in keratinocytes, and HO-2 in keratinocytes plays a role in maintaining iron release from ferritin^42^. The result that mild PAM did not exert preconditioning effects in A549 cells was attributed to the absence of an increase in HO-1 mRNA levels (Fig. 1h); however, the underlying mechanisms remain unclear.

HO-1 is positively regulated by the Keap1-Nrf2-ARE pathway^39^,^40^,^43^. In this study, we revealed the nuclear translocation (Fig. 3a and b) and recruitment of Nrf2 to *HO-1* promoters (Fig. 3c) in fibroblasts exposed to mild PAM for 6 h. The knockdown of Nrf2 attenuated the induction of HO-1 by mild PAM (Fig. 3e), suggesting that mild PAM up-regulates the Nrf2-dependent expression of HO-1. The up-regulation of HO-1 correlated with mild PAM-induced preconditioning effects against a high dose of H_2_O_2_ (Figs 1 and 2). Our results showed that the addition of ZnPP, a HO-1 specific inhibitor (Fig. 2f) or knockdown of Nrf2 (Fig. 3f) completely abrogated the preconditioning effects of mild PAM. H_2_O_2_-induced Keap1-Nrf2-ARE signaling pathway is well known^18^,^19^,^20^. In this pathway, H_2_O_2_ oxidize cysteine residues in Keap1 and change its conformation^18^,^44^. This conformational change weakens the interaction between Keap1 and Nrf2, and inhibits the subsequent ubiquitination and degradation of Nrf2. Therefore, Nrf2 is stabilized and translocated to the nucleus, and then binds to ARE in the *HO-1* promoter. The phosphatidylinositol 3 kinase (PI3K)-Akt signaling pathway is known to activate Nrf2^33^,^45^,^46^ and its downstream signaling induces HO-1^45^. H_2_O_2_ has been identified as an activator of the PI3K-Akt signaling pathway^46^.

ROS/RNS or their derived species are generally considered to be the main bioactive species in PAM. We speculated the involvement of other H_2_O_2_-cooperating species in PAM-induced apoptosis in cancer cells, whereas H_2_O_2_ needs to be the main active species in the reaction^25^. Moreover, ROS-derived amino acid peroxides (organic peroxides)^1^ and/or nitrites^36^ may contribute to the potency of PAM. It is not currently possible to rule out the involvement of these species and other unknown species and latent mechanisms in the preconditioning effects of mild PAM.

**Figure 4:**
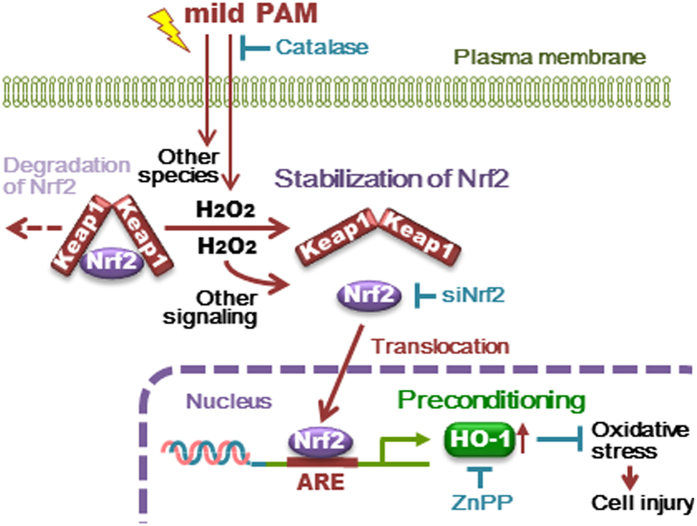
Cytoprotective effects of mild PAM against oxidative stress in human skin fibroblasts. Mild PAM protects fibroblasts from oxidative stress through the up-regulation of HO-1 mediated by the Nrf2-ARE pathway.

A relatively low dose of plasma has been reported to induce the proliferation of cells^7^,^8^, and has potential for clinical applications, for example, wound healing^2^,^47^,^48^. On the other hand, the induction of HO-1 has also been reported to play a crucial role in wound healing^41^,^49^,^50^. Our results clearly demonstrate that the pretreatment with mild PAM protected fibroblasts from oxidative stress by up-regulating HO-1; tens of μM of H_2_O_2_ in mild PAM contribute to preconditioning effects; the H_2_O_2_-induced activation of the Nrf2-ARE signaling pathway needs to be involved in the up-regulation of HO-1 (Fig. 4). Although further investigations are needed, the results of the present study provide evidence for the anti-oxidative stress functions of mild PAM and its potential for clinical applications.

## Materials and Methods

### Cell culture

Human skin fibroblasts were grown in Dulbecco’s modified Eagle’s medium (DMEM, Nissui Pharmaceutical Co., Tokyo, Japan) supplemented with 10% fetal calf serum (FCS), 100 units/mL penicillin, and 100 μg/ml streptomycin (DMEM-10%FCS) under an atmosphere of 5% CO_2_/95% air at 37 °C.

### Preparation of mild PAM

The experimental set-up of the NTAPP irradiation system used in this study consisted of a power controller/gas flow regulator, argon gas cylinder, and NTAPP source head (PN-120 TPG, NU Global, Nagoya, Japan) and was the same as the system described previously^25^,^26^,^27^,^28^. The flow rate of argon gas was set at 2 standard liters/min. Mild PAM was prepared by exposing NTAPP to 4 ml of DMEM (Sigma 5796), without FCS and antibiotics, in 60-mm culture dishes (Nunc 150288). The distance between the plasma source and surface of the medium was fixed at L = 4 mm. The duration time for PAM irradiation was 2 min, except in the time-course experiment.

### Assays to measure H_2_O_2_ and nitrite concentrations

H_2_O_2_ concentrations in mild PAM were assayed by a colorimetric method using 3-methyl-2-benzothiazolinone hydrazine hydrochloride, *N,N*-dimethylaniline, and horseradish peroxidase^25^. Nitrite as NOx was assayed by the Griss method^51^. The concentrations of H_2_O_2_ and nitrite in mild PAM were measured immediately after its preparation and it was promptly used in experiments.

### Cytotoxicity assay

The lactate dehydrogenase (LDH) assay was used to estimate cytotoxicity. Fibroblasts were seeded at 1.5 × 10^4^ cells/well in a 96-well microplate (Nunc 167008), cultured for 24 h in a CO_2_ incubator, and then used in experiments. Cells were treated with 80 μl of mild PAM for 6 h in a CO_2_ incubator. After the removal of mild PAM, cells were provided DMEM-10% FCS (80 μl) and incubated for 18 h in a CO_2_ incubator. After the removal of medium, cells were treated with 500 μM H_2_O_2_ in DMEM (80 μl) as an oxidative stress for 4 h in a CO_2_ incubator, and this was followed by an assay for LDH released into conditioned medium using a LDH cytotoxic test (Wako Pure Chemical, Osaka, Japan) according to the manufacturer’s directions.

### Reverse transcriptional-polymerase chain reaction (RT-PCR) analysis

Fibroblasts were cultured in 60-mm culture dishes (seeded at 4 × 10^5^ cells/dish) for 24 h in a CO_2_ incubator and then used in experiments. After the treatment with mild PAM (3 ml) for 6 h, cells were washed with cold phosphate-buffered saline (PBS) and total RNA was extracted from cells with TRIzol reagent (Invitrogen, Carlsbad, CA, USA). First-strand cDNA was generated from 1 μg of total RNA. RT-PCR was performed with specific primers described below and rTaq (Toyobo, Ootsu, Japan) according to the manufacturer’s directions. The primer sequences used in this study were as follows: HO-1, sense 5′-CCA GAA GAG CTG CAC CGC AA-3′, antisense 5′-GCT GGA TGT TGA GCA GGA AC-3′; HO-2, sense 5′-GAA GGA GCT GTT TAA GCT GG-3′, antisense 5′-GGG AGT TTC AGT GCT CGC TG-3′. Aliquots of PCR products were loaded on a 2% (w/v) agarose gel for electrophoresis, and a densitometric analysis of the PCR products was performed with Multi Gauge V3.0 (Fuji Film, Tokyo, Japan).

Real-time RT-PCR was performed using Thunderbird^TM^ SYBR qPCR Mix (Toyobo) according to the manufacturer’s protocol. The primers used for HO-1 were same as those described above.

### Western blotting

Fibroblasts were cultured in 60-mm culture dishes (seeded at 4 × 10^5^ cells/dish) for 24 h in a CO_2_ incubator and then used in experiments. After the treatment with mild PAM (3 ml) for 6 h in a CO_2_ incubator, cells were washed with cold PBS, scraped, and lysed in 50 μl of lysis buffer (20 mM Tris-HCl, pH 7.4, containing 1 mM EDTA, 1 mM EGTA, 10 mM NaF, 1 mM Na_3_VO_4_, 20 mM β-glycerophosphate, 1 mM phenylmethylsulfonyl fluoride (PMSF), 1 mM dithiothreitol (DTT), 2 μg/ml leupeptin, and 1% Triton X-100), followed by centrifugation at 17,000 × *g* for 5 min. After centrifugation, the protein concentration of the supernatant was assayed using a Bio-Rad protein assay reagent. Extracts containing 20 μg protein were boiled with sample buffer (62.5 mM Tris-HCl, pH 6.8, containing 2% sodium dodecylsulfate (SDS), 10% glycerol, 50 mM DTT, and 0.01% bromophenol blue) for 5 min and separated by SDS-PAGE on 15% (w/v) polyacrylamide gels. After being transferred electrophoretically onto PVDF membranes, non-specific binding sites were blocked with PBS containing 1% bovine serum albumin (BSA). Membranes were subsequently incubated with an anti-HO-1 antibody (1:1,000, Cell Signaling Technology, Danvers, MA, USA) or anti-β-actin antibody (1:1,000, Merck Millipore, Billerica, MA, USA). After the membranes had been washed three times with PBST (PBS containing 0.1% Tween 20), the blots were incubated with biotin-conjugated goat anti-rabbit or -mouse antibody (1:1,000, Zymed Laboratories, South San Francisco, CA, USA), followed by an incubation with ABC reagents (1:5,000, Vector Laboratories, Burlingame, CA, USA). Bands were detected using ImmunoStar LD (Wako Pure Chemical), and imaged using an LAS-3000 UV mini (Fuji Film).

Regarding nuclear extraction, fibroblasts were collected and lysed in 800 μl of lysis buffer A (20 mM HEPES-NaOH, pH 7.8, containing 15 mM KCl, 2 mM MgCl_2_, 2 mM DTT, 0.5 mM PMSF, and 10 μg/ml leupeptin) followed by centrifugation at 830 × *g* for 30 sec. The pellets were suspended in 150 μl of lysis buffer B (lysis buffer A containing 0.5% Nonidet P-40 (NP-40)) and incubated for 30 min on ice. After centrifugation at 9,000 × *g* for 30 sec, the pellets were suspended in 40 μl of lysis buffer C (20 mM HEPES-NaOH, pH 7.8, containing 0.4 M NaCl, 2 mM DTT, 0.5 mM PMSF, and 10 μg/ml leupeptin). After centrifugation at 20,000 × *g* for 20 min, the supernatant was saved as the nuclear fraction. The protein concentration of the supernatant was assayed using a Bio-Rad protein assay reagent. Extracts containing 20 μg protein were boiled with sample buffer for 5 min and separated by SDS-PAGE on 12% (w/v) polyacrylamide gels. After being transferred electrophoretically onto PVDF membranes, non-specific binding sites were blocked with PBS containing 1% BSA. Membranes were subsequently incubated with an anti-Nrf2 rabbit polyclonal antibody (1:1,000, Santa Cruz Biotechnology, Santa Cruz, CA, USA) or anti-histone H3 rabbit monoclonal antibody (1:1,000, Cell Signaling Technology). After the membranes had been washed three times with PBST, blots were incubated with a biotin-conjugated goat anti-rabbit or -mouse antibody (1:1,000), followed by an incubation with ABC reagents (1:5,000). Bands were detected using ImmunoStar LD, and imaged using an LAS-3000 UV mini.

### Fluorescence imaging for Nrf2

Fibroblasts (6 × 10^4^ cells) were seeded on collagen-coated coverslips (12 mm in diameter) in a 4-well culture plate (Nunc 176740) and cultured for 24 h in a CO_2_ incubator. After the treatment with mild PAM (500 μl) for 6 h in a CO_2_ incubator, cells were washed with PBS followed by fixation with 3% paraformaldehyde, permeabilization with 0.1% Triton X-100, and blocking with 3% BSA solution. Cells were then incubated for 1 h with an anti-Nrf2 antibody (1:50) diluted with Can Get Signal Immunostain solution (Toyobo), followed by an incubation for 1 h with Alexa Fluor 488 goat anti-rabbit IgG (1:400). After the labeling of cell nuclei with Hoechst 33342 (1:1,000, Dojindo, Kumamoto, Japan), cells were washed and visualized under the LSM710 confocal laser fluorescence microscope (Carl Zeiss, Gottingen, Germany).

### Chromatin Immunoprecipitation (ChIP) Assay

Fibroblasts seeded at 1 × 10^6^ cells/dish in a 90-mm culture dish (Nunc 150350) were cultured for 24 h in a CO_2_ incubator. After the cells had been treated with mild PAM (10 ml) for 6 h in a CO_2_ incubator, protein-DNA complexes were cross-linked using 1% formaldehyde in DMEM-10%FCS at room temperature for 5 min. The reagent was subsequently quenched by the addition of 200 mM glycine in DMEM at room temperature for 5 min. Cells were washed with PBS, and then scraped and lysed in 1 ml of NP-40 buffer (10 mM Tris-HCl, pH 8.0, containing 10 mM NaCl and 0.5% NP-40), and centrifuged at 830 × *g* for 3 min. The pellet was dissolved in 100 μl of SDS buffer (50 mM Tris-HCl, pH 8.0, containing 1% SDS and 10 mM EDTA), and added to 400 μl of ChIP dilution buffer (50 mM Tris-HCl, pH 8.0, containing 167 mM NaCl, 1.1% Triton X-100, 0.11% deoxycholic acid, 10 mM NaF, 1 mM Na_3_VO_4,_ 20 mM β-glycerophosphate, 1 mM DTT, and 1 mM PMSF). Genomic DNA was sheared using an ultrasonic homogenizer Vivracell VC100 (Sonic & Materials, Danbury, CT, USA) to achieve an estimated DNA size range of 150 to 800 bp, and 500 μl of ChIP dilution buffer was then added. One hundred microliters of sheared genomic DNA was diluted with 400 μl of RIPA buffer I (50 mM Tris-HCl, pH 8.0, containing 150 mM NaCl, 1 mM EDTA, 0.1% SDS, 0.1% deoxycholic acid, and proteinase inhibitors) and incubated with a rabbit anti-Nrf2 antibody (1:100) at 4 °C overnight with shaking. The solution was subsequently incubated with 20 μl of Dynabeads Protein G (Invitrogen) at 4 °C for 2 h with shaking. After being incubated, the beads were sequentially washed with RIPA buffer I, RIPA buffer II (50 mM Tris-HCl, pH 8.0, containing 500 mM NaCl, 1 mM EDTA, 0.1% SDS, 0.1% deoxycholic acid, and proteinase inhibitors), and TE buffer (10 mM Tris-HCl, pH 8.0, containing 1 mM EDTA). The beads were suspended in 100 μl of ChIP elution buffer (10 mM Tris-HCl, pH 8.0, containing 300 mM NaCl, 5 mM EDTA, and 0.5% SDS) with 1 μl RNase (Roche Diagnostics, Mannheim, Germany) and incubated at 37 °C for 30 min, followed by the addition of 1 μl proteinase K (Roche Diagnostics) and an incubation at 65 °C for 2 h. After phenol-chloroform extraction and ethanol precipitation, genomic DNA was dissolved in 20 μl of TE buffer. The abundance of ARE in ChIP precipitates was quantified using a RT-PCR analysis. The primer sequences for ARE were sense 5′-CCC TGC TGA GTA ATC CTT TCC CGA-3′, antisense 5′-ATG TCC CGA CTC CAG ACT CCA-3′.

### RNA interference study

Nrf2-specific short interfering RNA (siRNA) and scramble control siRNA were obtained from Life Technologies Japan (Tokyo, Japan). Transfection was performed using Lipofectamine RNAiMAX Reagent (Thermo Fischer Scientific, Yokohama, Japan), according to the manufacturer’s protocol with Nrf2-specific siRNA AM16708; human NFE2L2; target sequences including sense 5′-CCU UAU AUC UCG AAG UUU Utt-3′; antisense 5′-AAA ACU UCG AGA UAU AAG Gtg-3′. Briefly, cells were transfected with 50 nmol/l siRNAs directed against Nrf2 (siNrf2) or negative control siRNA (siNC) and then cultured for 24 h in a CO_2_ incubator, followed by assays for HO-1 mRNA levels and cytoprotective ability.

### Data analysis

Data are presented as the mean ±SD of three experiments. Data were analyzed by Welch’s *t*-test. A *p* value of less than 0.05 was considered significant.

## Additional Information

**How to cite this article**: Horiba, M. *et al*. Cytoprotective effects of mild plasma-activated medium against oxidative stress in human skin fibroblasts. *Sci. Rep.* **7**, 42208; doi: 10.1038/srep42208 (2017).

**Publisher's note:** Springer Nature remains neutral with regard to jurisdictional claims in published maps and institutional affiliations.
